# 

**Published:** 1901-10-26

**Authors:** 


### NOTICE TO CORRESPONDENTS.

All MSS., Letters, Books for Review, and other matters intended for the Editor should be addressed The Editor, "The Hospital," The Hospital Building, 28 & 29 Southampton Street, Strand, W.C.

The Editor cannot undertake to return rejected MS., even when accompanied by stamped directed envelope.

**Notice to Advertisers and Subscribers.**

All Advertisements, Orders for Copies of The Hospital, and Business Communications should be addressed to **THE MANAGER (Not to the Editor),** The Hospital, 28 & 29 Southampton Street, Strand, London, W.C.

The Cost of Subscription, post free, is as follows:—Per week, 2½d.; per quarter, 2s. 8d.; per half-year, 5s. 3d.; per annum, 10s. 6d. Foreign :— Per annum, 15s. 2d., payable, in all cases, in advance.

**NOTICE.—Vol. XXX. of "The Hospital" (April 1901, to September 1901), in handsome cloth binding will shortly be on sale at the Office of this Journal, price, 7s. 6d. Cases for binding the Numbers contained in this Volume 1s. 6d. Index to Vol. XXX., 2½d., post free.**

**The Monthly Part for October is now ready. Price 6d., by post 8d.**

### BOOKS RECEIVED.

Rebman, Limited.

"International Directory of Laryngologists and Otologists," containing names and addresses of practitioners engaged in the study and practice of laryngology and otology. Compiled by Richard Lake, F.R.C.S.

Edward Arnold.

"Phototherapy." By Professor Niels R. Finsen, translated from the German edition; and with an Appendix on the Light Treatment of Lupus. By James H. Sequeira, M.D., Lond. M.R.C.P.

Swan Sonnenschein and Co.

"Psychology, Normal and Morbid." By Charles Mercier, M.B., M.R.C.P., F.R.C.S.

### SCRAPS AND GLEANINGS.

The Jessop Hospital for Women, Sheffield, is expending upwards of £20,000 on much needed extensions in various departments of the hospital.

The total amount drawn at the three days' bazaar held at Perth in aid of the Perth Consumptive Hospital, together with donations, was £4,703.

The Lord Mayor's fund for the National Memorial to Queen Victoria now amounts to £56,200. Included in this total is a sum of £1,524 from the city of Birmingham.

The net receipts this year at the Eastbourne Church Parade and Cycle Collection, on behalf of the Princess Alice Hospital, were £72, being an increase of £11 over last year.

With a view to checking the inroads which the Americans are making upon the optical trade of the United Kingdom the Optical Society has appointed a committee to inquire into the system at present in operation at the Northampton Institute, Clerkenwell, under which classes in optical subjects are held there, and to consider the feasibility of approaching the various institutions in London and the provinces with a view to the establishment of similar classes.

### THE STUDENT OF MEDICINE.

### APPOINTMENTS.

**D. M. Alexander**, M.B., appointed Assistant Medical Officer of the Nottingham Workhouse Infirmary. **W. F. Victor Bonney**, M.D., M.S.Lond.. F.R.C.S., M.R.C.P., appointed Physician to Out-patients at the Chelsea Hospital for Women, and Physician-Accoucheur to the St. Pancras and Northern Dispensary. **A. H. Bygott**, L.S.A., appointed Medical Officer and Public Vaccinator for the Bordesley District of the Aston Union. **Annie F. M. Cornall**, L.R.C.P.S.Edin., L.F.P.S.Glasg., appointed Medical Officer to the Lady Kinnaird Memorial Hospital Zenana Medical Mission, Lucknow. **B. W. Gowring**, M.R.C.S., L.R.C.P., appointed District Medical Officer of the Basingstoke Union. **A. F. Griffiths**, M.D.Harvard, appointed a Clinical Assistant at the Chelsea Hospital for Women. **A. J. Harrison**, M.B.Lond., appointed Physician in charge of the Skin Department of the Bristol General Hospital. **J. F. Hodgson**, M.B., Ch.B.Vict., appointed Resident Medical Officer of the Halifax Union Poor-law Hospital. **E. J. McCardel**, M.D., Ch.M., M.R.C.S.Eng., appointed a Clinical Assistant at the Chelsea Hospital for Women. **Roderick M. Matheson**, M.D., M.S.Edin., appointed Honorary Assistant Surgeon to Noble's Isle of Man Hospital. **Newman Neild**, M.B., Ch.B.Vict., appointed Assistant Physician to the Bristol General Hospital. **George Parker**, B.A.Camb., M.D., appointed Physician to the Bristol General Hospital. **R. Craig Rodgers**, M.R.C.S.Eng., L.R.C.P.Lond.. appointed Honorary Medical Officer to the Burnley Victoria Hospital. **C. Stanley Steavenson**, M.B., B.Ch., appointed Senior House Surgeon to the Kent and Canterbury Hospital. **Wm. Sutcliffe**, M.R.C.S., L.R.C.P., L.S.A., appointed Medical Officer of No. 3 District of the West Bromwich Union. **A. H. Turner**, L.S.A., appointed Medical Officer and Public Vaccinator for the Beaconsfield District of the Amersham Union. **Willy Von Muralt**, M.D.Zurich, appointed a Clinical Assistant at the Chelsea Hospital for Women. **D. J. G. Watkins**, M.B., B.C.Cantab., M.R.C.S.Eng., appointed Honorary Surgeon to the Lincoln County Hospital.

**An ATLAS of BACTERIOLOGY.**

Containing 111 Original Photo-Micrographs, with Explanatory Text.

By CHARLES SLATER, M.A., M.B., M.R.C.S.Eng., F.C.S., and EDMUND J. SPITTA, L.R.C.P.Lond., M.R.C.S.Eng.

Demy 8vo., cloth gilt, 7s. 6d. net.

"It is impossible to deny that this work fills a blank in the life of the student of bacteriology. The photographs are excellent and the letterpress, linking together and explaining the teaching of the illustrations, is clear, concise, and accurate."—*Nature.*

The Scientific Press, Ltd., 28 & 29 Southampton Street, Strand, London, W.C.

